# Age and Body Size of the Endemic and Critically Endangered Frog Species *Rana tavasensis* (Baran and Atatür, 1986) in Türkiye

**DOI:** 10.3390/ani14182703

**Published:** 2024-09-18

**Authors:** Ufuk Bülbül, Eyup Başkale, Hatice Özkan

**Affiliations:** 1 Department of Biology, Faculty of Science, Karadeniz Technical University, 61080 Trabzon, Turkey; haticeozkan@ktu.edu.tr; 2 Department of Biology, Faculty of Science, Pamukkale University, 20070 Denizli, Turkey; ebaskale@pau.edu.tr

**Keywords:** longevity, skeletochronology, Tavas, SVL, species conservation, Türkiye

## Abstract

**Simple Summary:**

The main objective of this study was to provide the first information on the age structure of the critically endangered and endemic frog species, *Rana tavasensis*, in Türkiye. Age data are important in evaluating the dynamics of populations and effective conservation strategies should be addressed according to age-related parameters (e.g., longevity, mean age, growth rate, and age at maturity). Skeletochronology is an effective method for estimating the ages of amphibians, and using this method, we determined the mean age, longevity, and relationship between age and body size in both males and females of a *R. tavasensis* population in Çakıroluk, the terra-typica, located in Denizli province, Türkiye. The results of our study provides insights for taking feature measures to protect these frogs, whose number has progressively declined each year due to habitat loss and degradation.

**Abstract:**

This study used skeletochronology to assess the relationships between age and body length among individuals in a population of the Tavas Frog (*Rana tavasensis*), located in the Çakıroluk plateau, Tavas district, Denizli province. The age varied from 3 to 12 years in both adult males and females. The age was 2 years in two subadult males, while it was 1 year in one juvenile specimen. The mean SVL and age of the adult individuals of the Çakıroluk population were 62.75 mm and 6.70 years in male specimens and 58.04 mm and 6.12 years in female specimens. A positive correlation was found between body size (SVL) and age in adult female and male individuals of the species. Because the number of individuals of the species is rapidly decreasing, species protection measures, based on knowledge related to the age structure and breeding features of these frogs, must be put into action urgently.

## 1. Introduction

The Tavas Frog, *Rana tavasensis*, is an endemic species in Türkiye, first described in 1986 [1] as a new subspecies (*Rana macrocnemis tavasensis*) of the Long-Legged Wood Frog (*Rana macrocnemis*) in the Çakıroluk area, Denizli, Türkiye. Later, it was raised to the species level based on differences in partial sequences of 16S rRNA gene [2]. A second population of *R. tavasensis* was reported [3] in Lake Girdev in Muğla province, Türkiye based on Max Kasparek’s personal observations and not on scientific evidence. In addition, five individuals of the species were reported in the Atlıdere district of Muğla province, located 30 km north of Lake Girdev [4]. *Rana tavasensis*, which is classified in the CR category according to IUCN, is very rare and found in only two distinct locations. In 2011, the population size of Çakıroluk was estimated to be 398 mature individuals [5]. This number has progressively declined each year due to habitat loss and degradation, reaching 48 in 2018, representing a population reduction of 88%. A recent monitoring study in Çakıroluk found that the population size of mature individuals decreased from 85 to 39 between 2011 and 2018. This new study, along with subsequent observations, indicates that almost 46% of mature individuals have disappeared in the past decade [5,6]. There is no scientific data on the decline in the number of individuals in the Girdev Lake population over time or on the mortality rates of adult individuals. After a survey effort of eight person-hours, only eight juveniles and no mature individuals were found [6]. Nevertheless, conducting a survey lasting only a few hours is inadequate for assessing population decline or making any population estimates. In Girdev Lake, it is estimated that the population contains fewer than 250 mature individuals. Due to the continuous decline in the extent and quality of its habitat, the population is understood to be decreasing [6].

In conservation biology, knowledge of the age structure is critical because it aids in understanding the ecological and biological needs required for protecting endangered species. Age data assists in monitoring population dynamics (whether it consists mostly of young or old individuals), growth rates, longevity, sexual maturity, the duration of active reproductive periods, and reproductive success, all of which are critical for devising an effective conservation strategy [7,8].

Skeletochronology is an effective method for obtaining information on the age of amphibians [9]. It can be utilized on amphibian phalanges, allowing for sampling without the need for animal culling and without any negative impact on amphibian populations or endangered species [10,11,12,13,14]. While age determination studies exist for *Rana* species in Türkiye (*Rana dalmatina* [15,16], *Rana holtzi* [11,17,18], and *Rana macrocnemis* [19]), no such studies have been conducted for *Rana tavasensis*. In this context, we focus on providing the first information on the age structure of individuals from the Çakıroluk population. Thus, we present for the first time the mean age, age at sexual maturity (the minimum number of lines of arrested growth counted in reproductive individuals), longevity (the maximum number of LAGs counted in reproductive individuals), and potential reproductive lifespan (the difference between longevity and age at maturity) as indicated in the current literature [20].

## 2. Materials and Methods

**Figure 1:**
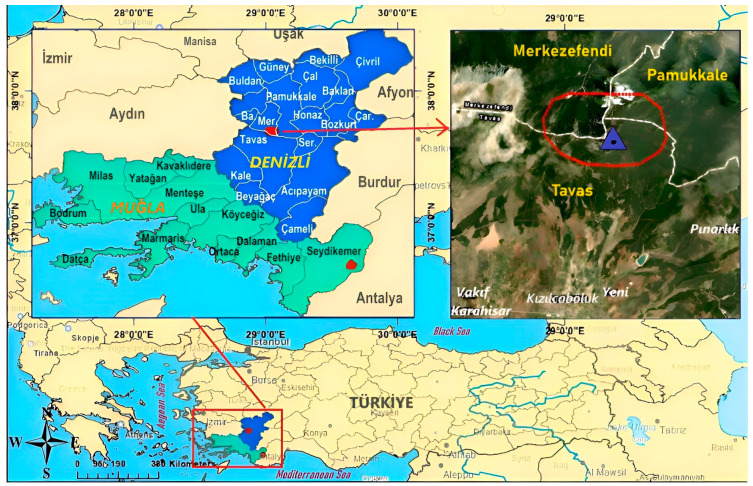
The distribution area of *Rana tavasensis* according to IUCN (which is shown with red colored dots in Denizli and Muğla provinces) and the Çakıroluk locality (where the samples were collected) in the Tavas district of Denizli province. The blue triangle shows study area in Çakıroluk.

In total, 40 individuals of *Rana tavasensis* (18 ♂♂, 18 ♀♀, 2 subadult ♂♂, and 2 juveniles) were caught between 28 June and 23 August 2019 in the Çakıroluk locality in the Tavas district of Denizli province, Türkiye. However, we obtained usable results from 10 ♂♂, 17 ♀♀, 2 subadult ♂♂, and 2 juveniles in the skeletochronological analyses due to problems with decalcification. The study site (37.6907 K, 29.0493 E) was located in a mountainous area at 1648 m a.s.l. (Figure 1).

The habitat of the frogs consists of meadows located in a pine forest (*Pinus nigra*) with small temporary water bodies. The area where the species is distributed has subalpine vegetation. 

Çakıroluk, situated in the elevated and mountainous region of Denizli province in the Inner Aegean, has a continental climate typical of Central Anatolia. Consequently, winters are notably severe, with cold temperatures and abundant snowfall, while summers remain cool. The temperature is below zero in December, January, and February. The climate data covering the last three decades of the Çakıroluk site were provided by the Turkish Ministry of Environment, Urbanization and Climate Change, General Directorate of Meteorology. The mean annual temperature and precipitation are 16.3 °C and 47.44 mm per square meter, respectively. The active period of the Çakıroluk frog population extends from early April to the end of October. During the active period of the frogs, the mean temperature and precipitation are 21.8 °C and 28.67 mm, respectively. 

The snout vent lengths (SVLs) were measured with a digital caliper (Mitutoyo Absolute Digimatic, Kanagawa, Japan). The frogs were caught by hand and sexes were then determined by direct examination of the sexual characters (presence of gray-colored nuptial pads on the first fingers of the forelimbs of the male individuals during the breeding season). We observed the frogs during a day’s excursion between 9.30 a.m. and 7.00 p.m. The air temperature was 23 °C during the observation time. We classified the life stages of *Rana tavasensis* based on the SVL measurements and the reproductive behavior of individuals during field studies because the species is both critically endangered and endemic, and there is limited information available on the population biology of the species. We collected individuals who exhibited reproductive behavior, such as sexual attraction to the opposite sex and couples in the amplexus as adults. All adult males had developed nuptial pads on the first finger of their front foot. We collected two individuals with very small nuptial pads who did not display reproductive behavior, were relatively far from reproductive areas during the sampling period, and had a snout vent length (SVL) ranging from 42.73 to 50.40 mm, classifying them as subadult males. The two individuals, which appeared very small and had a snout-vent length (SVL) ranging from 16.95 to 29.89 mm, were classified as juveniles. According to the standard skeletochronological technique [21] used to determine the age of an individual animal, the second phalanx of the longest toe of the hind foot was cut and all samples were preserved in 10% formalin solution until laboratory studies. After the sex determination, recording of body length measurement data, and toe-clipping, the frogs were released back into their natural habitats. The animals were treated in accordance with the guidelines of the Ethics Committee of the Karadeniz Technical University 

The fixed toes were processed following the routine skeletochronological procedure [21,22,23,24,25]. All finger bone samples were cleared of soft tissues, washed in running water for 12 h, decalcified for 4–16 h (depending on the size of the bones) in 5% nitric acid, and then placed in distilled water overnight. All toe samples were loaded into a tissue processing system (Leica TP1020 tissue processor, Wetzlar, Germany). Later, all tissue samples were embedded in paraffin with a tissue embedding device (Thermo, Waltham, MA, USA). Each bone was sectioned from the central region of the diaphysis using a rotary microtome with a thickness of 10 μm. The cross-sections were stained with hematoxylin and were then observed under a light microscope (ZEISS Primostar, Oberkochen, Germany) and photographed with the aid of a microscope camera (ZEISS Axio Cam ERc 5s, Oberkochen, Germany). The number of lines of arrested growth (LAGs) in each of the bone cross-sections was observed and counted independently by two authors (Ufuk Bülbül and Hatice Özkan) to minimize the error rate.

LAGs form annually; therefore, each LAG may represent a year of life [21]. Sometimes, though not always problems may arise where double lines can be perceived as if they were two real LAGs. This is because the species experiences two periods of arrested growth in the same year. Nevertheless, many environmental influences (seasonal changes related to the climate; the spring summer period of growth is recorded by the wide band of bone tissue and the autumn-winter cessation of growth is recorded by the resting line) impacting LAG growth occur within a year [26]. In this study, we evaluated the double line as a single LAG, like most studies in the literature [11,27,28]. In addition to the LAGs, one ring on the innermost side was interpreted as a metamorphosis line (ML). LAGs and MLs can be easily distinguished by their different shapes, as described in the literature [29].

The *psych* package v. 2.4.6.26 [30] was used to summarize the statistical data, present the basic characteristics of the measurements, and calculate descriptive statistics. The normality of the variables was assessed using the Shapiro Wilk test, which is robust for small sample sizes [31]. Since age and SVL were not normally distributed (Shapiro Wilk test, *p* < 0.05), the Mann Whitney U test, a nonparametric test, was used to compare the variables between sexes. Spearman’s correlation coefficient was then calculated to evaluate the relationship between SVL and age. 

Sexual size dimorphism (SSD) was quantified using the size dimorphism index (SDI) as described in the literature [32]. SDI = (mean length of the larger sex/mean length of the smaller sex) ± 1. This formula assigns a value of +1 if males are larger than females, resulting in a negative SDI, or −1 if females are larger than males, resulting in a positive SDI.

Growth was estimated according to the von Bertalanffy equation [33] which was previously used in several studies on amphibians [34,35,36,37]. The modified growth formula is; 

SVLt = SVLmax − (SVLmax − SVLmet)e − k(t − tmet) where SVLt is the average body length at age t; SVLmax is the asymptotic maximum body length; SVLmet is the body length at metamorphosis, which was used to calculate newly metamorphosed individuals (*n* = 14), that were caught at the end of the summer (28–30 August 2024) (fixed to the mean = 12.7 ± 1.297 mm for the Çakıroluk population); k is the body growth rate coefficient (units are yr ^− 1^) that defines the shape of curve; tmet is the age at metamorphosis (0.3 yr ^− 1^).

The survival rates assume a constant survival rate across all age classes and sampling of individuals with respect to age, which were estimated from Robson and Chapman’s formula [38]: S = T/(R + T − 1) where S is the finite annual survival rate estimate, T is N1 + 2N2 + 3N3 +…. and R is ƩNi, where Ni is the number of individuals in age group i.

Adult life expectancy (ESP) is the expected average age and differs from the longevity value, which is the highest recorded age amongst individuals. Adult life expectancy (ESP) was derived from Seber’s formula [39]: ESP= 0.5 + 1 /(1 − S), where S is the survival rate.

The statistical analyses were performed using the *stats* v. 4.4.0 package. The visualization of data was performed utilizing the *ggstatsplot* [40] package v. 0.12.4. All analyses were conducted using the R programming language v. 4.4.1 [41].

## 3. Results

**Figure 2:**
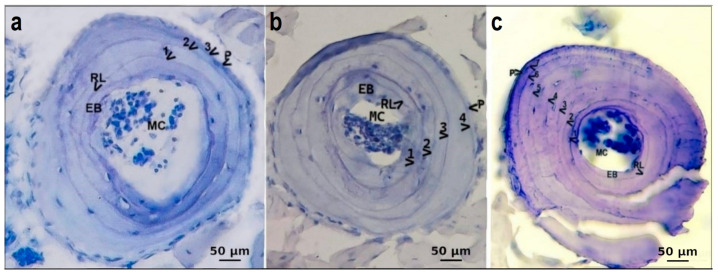
(**a**) A cross-section (10 µm thick) through the phalange of a three-year-old male (51.13 mm SVL) *Rana tavasensis*. (**b**) A cross-section (10 µm thick) through the phalange of a four-year-old female (49.87 mm SVL) *Rana tavasensis*. (**c**) A cross-section (10 µm thick) through the phalange of a seven-year-old male (66.16 mm SVL) *Rana tavasensis*. For abbreviations, see text (MC: marrow cavity; EB: endosteal bone; RL: resorption line; and P: periphery). Periphery was not counted as a LAG.

By comparing the diameters of the eroded marrow cavities of the adults with those of the non-eroded marrow cavities of the juveniles, we determined that endosteal resorption did not destroy any LAGs or cause any problems in determining the age of a frog. Although the diameter of the medullary cavity of the adults (measured with an ocular micrometer) varied, it never exceeded that of the subadults. In all preparations, the resorption zone was clearly visible outside the endosteal bone, posing no challenges for age determination. A growth zone and a thin hematoxylinophilic outer line corresponding to a winter line of arrested growth were present in the cross-sections of the phalanges of all individuals. (Figure 2). We observed ML only in some of the younger individuals, while it was not present in the older individuals. Out of all the examined adult individuals, 16 (59.3%) showed endosteal resorption, and 11 (40.7%) showed double LAGs.

We found that the subadult males were 2 years old, while the juvenile specimens were 1 year old. Both the adult males and females ranged in age from 3 to 12 years. We estimated the potential reproductive lifespan for both sexes to be 9 years.

Age and SVL measurement values are given in Table 1.

We found that both the adult males and females of *R. tavasensis* in the Çakıroluk population were between 3 and 12 years old. All these individuals came to the water to breed, and their body lengths were between 51.13 and 71.69 mm for males and 42.33 and 73.97 mm for females. We considered these individuals to be adult individuals. Therefore, the age of sexual maturity for both sexes in this population can be considered to be three years. However, it is necessary to check whether their gonads are developed to determine the exact age at which the frogs reach sexual maturity. Since this species is critically endangered, we did not carry out the procedure, as it would risk causing the death of the frogs. 

The means of the SVL and age were, 59.78 ± 8.41 mm and 6.33 ± 2.63 years for all adults individuals of *Rana tavasensis* (62.75 ± 8.02 mm and 6.70 ± 2.90 years in the male specimens and 58.04 ± 8.37 mm and 6.12 ± 2.52 in female specimens), respectively (mean ± SD) (Table 1).

The mean ESP of the females and males was estimated to be 8.19 and 9.17 years, respectively. Similarly, the mean survival rates of the females and males were calculated to be 0.87 and 0.88, respectively.

**Figure 3:**
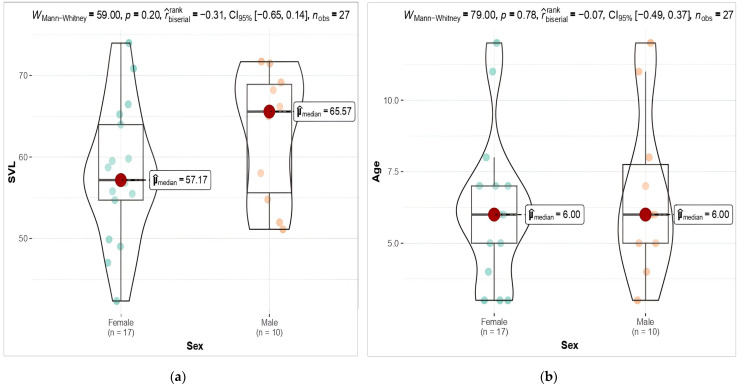
(**a**) Plots represent SVL differences between sexes (M: male, F: female); (**b**) Plots represent SVL differences between sexes (M: male, F: female). The associated *p*-values are shown on the relevant plots.

The intersexual difference in body size (length) was male-biased (SDI = −2.081). However, the mean SVL (Mann Whitney U-Test; 59, P = 0.20) and the average age (Mann Whitney U-Test; 79, P = 0.78) showed no statistical differences between the sexes (Figure 3). 

There was a correlation between age and SVL in both the males [Spearman’s correlation coefficient ρ = 0.70, p = 0.02, S = 49.30, CI95% (0.11, 0.93), *n* = 10] and females [Spearman’s correlation coefficient ρ = 0.89, p = 2.05 × 10 ^−6^, S = 92.02, CI95% (0.70, 0.96), *n* = 17].

**Figure 4:**
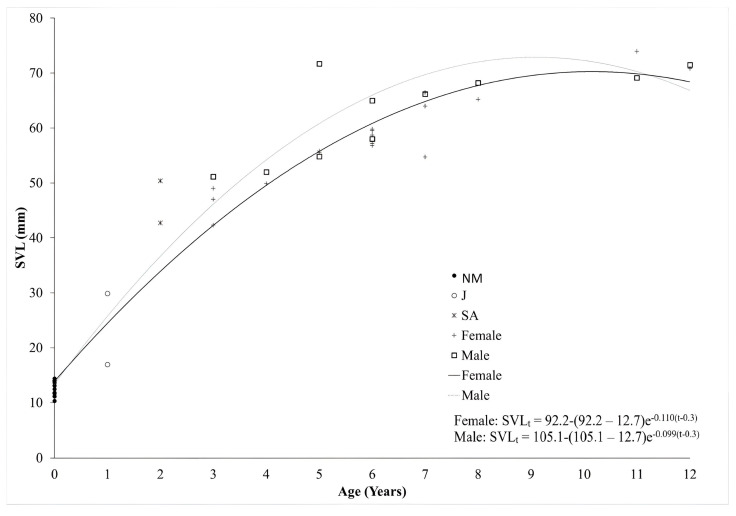
The von Bertalanffy growth curves for males and females of *R. tavasensis*. NM: newly metamorphosed, J: juvenile, SA: subadults.

Furthermore, the male and female growth patterns of the Çakıroluk populations were compared according to the von Bertalanffy growth model (Figure 4). We found that the growth coefficients were higher in the males than in the females, while the peak growth rate was found to be in the age range of 1–3 years. This slowly decreased after that to the age range of 3–4 years in both sexes.

## 4. Discussion

The present study provides the first data on age-related parameters, including longevity, reproductive life-span, and mean age, as well as the relationship between age and SVL. The longevity was found to be 12 years in both males and females of the Çakıroluk population (located at 1648 m a.s.l.) of *Rana tavasensis*. Previous studies pointed out the maximum age of brown frogs increased with an increasing latitude or altitude [42,43,44,45]. Similar to this general expectation, the maximum age was found to be relatively low (4–8 years) in Agile Frog (*Rana dalmatina*) populations; these frogs live at low altitudes in Italy, Spain, and Türkiye. In addition, it was found that the maximum age of *Rana macrocnemis* increased with altitude [5 years in Maçka (350 m a.s.l.), 6 years in Hıdırnebi (1450 m a.s.l.), 8 years in Sarıkamış (2276 m a.s.l.) and 10 years in Ovit (2850 m a.s.l.)] in Turkish populations of the species [19]. A similar trend with some minor differences [6 years at 850 m a.s.l., 7 years at 1950 m, 9 years at 1800 m, 11 years at 2600 m, and 12 years at 2400 m] was reported in Georgian populations of *R. macrocnemis* [46]. 

The longevity of other brown frog species varies depending on species (genetic) or factors in different environments of the same species (environmental) (Table 2).

Species and populations with longer lifespans have more reproductive periods, potentially leading to higher reproductive success. Specimens of *R. macrocnemis* living in the forest zone at 850 m a.s.l. typically have 3 reproductive periods, while those in the mountain population of Armenia can have up to 9–10 periods. These factors must be considered when developing measures for species conservation [47]. We found a relatively higher potential reproductive lifespan (9 years) in the Çakıroluk population of the critically endangered frog species, *R. tavasensis*. This potentially provides an advantage, with individuals in this population having more chances to reproduce. However, many amphibian species are subjected to the impacts of two factors: spring floods and droughts. As a result of spring floods, many shallows form at a distance from the primary spawning areas. Due to their preference for certain water depths and temperatures, many frogs breed in small temporary water bodies that disappear quickly after reproduction [47]. Our field observations also show that this situation negatively affects the development of the eggs and larvae of *R. tavasensis*. Adult individuals protect themselves by hiding in crevices in the ground during the summer months when the air temperature exceeds 20 °C [47].

Although we found a male-biased SSD, the differences in the mean SVL between the sexes of *R. tavasensis* were not significantly important. This may be due to the small sample size. Similar to our results, males and females of *R. macrocnemis* did not exhibit any differences in terms of the mean SVL in all populations, except for Sarıkamış where the males were significantly larger than the females [19]. In *R. holtzi*, the mean SVL did not differ between sexes in two studies [11,18], while males were found to be significantly larger in another study [17]. The fact that males were found to be slightly larger than females in Anatolian mountain frogs but without a statistically significant difference may be due to insufficient sampling. Since the factors affecting the body size of males or females in each population (such as food abundance, competition, male male combat, and temperature) are specific to the population, it is important to examine a large number of samples in order to clearly determine the size differences between the sexes in those populations. Similar to body size, the differences in mean age were not found to be significant between the sexes of *R. tavasensis*, *R. macrocnemis* and *R. holtzi*. However, a positive correlation was observed between age and SVL for both sexes in all populations of Anatolian mountain frogs, with a few exceptions. 

In our investigation, we detected double lines in 11 (40.7%) adult specimens. The double lines may form due to a secondary period of arrested growth, or sudden climatic changes [22,48,49]. Unpredictable ecological factors, such as fluctuations in food availability and occasional environmental changes, can lead to unexpected variations in double lines [11,50]. We observed a relatively high rate (40.7%) of double ring formation in adult specimens from the Çakıroluk population of *R. tavasensis*. The frogs live in unfavorable conditions (in this mountainous region, the climate is cold and snowy in winter and hot and dry in summer). Furthermore, environmental conditions can influence endosteal resorption, and elevation may lead to changes in this process [51]. In accordance with this, we found a high rate of (59.3) endosteal resorption in adult specimens of the Çakıroluk population. 

Our results indicating the ages of juvenile, subadult, and adult individuals of *R. tavasensis* in Çakıroluk show that the frogs do not mature early (in 1 or 2 years) and that the potential reproductive lifespan is not short (9 years) in both sexes. This situation offers individuals in this population the chance to mature with a larger body size, greater fertility, and more years of reproduction. In temperate zones, anurans, which mature later at a significantly larger size and age, have greater fecundity throughout their life [52]. Despite these advantages, the fact that the number of frogs in the Çakıroluk population has decreased significantly according to the current literature, suggests measures should be taken to protect the individuals in this population without delay. According to the species conservation and action plan prepared in 2019 [47], the factors threatening the Tavas Frog in the Çakıroluk population are as follows: habitat loss and fragmentation, grazing pressure by sheep and cattle, water intake from the Çakıroluk spring (for the purpose of creating fire pool water and using the water in animal husbandry activities in the area), pollution, motor vehicles and cars (adult and young individuals of the species are crushed to death due to off-road and motocross activities near and within their habitat), and the excessive collection of the frogs for scientific purposes.

The conservation measures outlined in the species conservation and action plan to improve the status of the species are as follows: 

(1) The protection of the habitat and ecological environment features of the Tavas Frog (the protection of existing habitats, prevention and elimination of forest fires, regulation of forestry activities, control of water intake from the Çakıroluk spring, regulation of motor vehicle traffic near the natural habitat of the species, and declaration of new protected areas).

(2) The monitoring, protection, and determination of success criteria for population-based species protection activities (preventing the decrease in the number of individuals by monitoring the population).

(3) Carrying out in situ rehabilitation studies.

(4) The education of local people, raising the awareness level and creating a sense of responsibility (conducting information and awareness studies, and ensuring coordination with stakeholders) [47]. Currently, the area where the species lives has been fenced, is protected by official authorities and, monitored with camera traps, and has warning signs.

## 5. Conclusions

Our study provides preliminary knowledge on the life history traits (e.g., the mean age, longevity, and the relationship between body size and age) of a critically endangered mountain frog species in Türkiye. Field observations of the Çakıroluk population indicate a significant decline in the number of individuals, serving as a crucial warning for this critically endangered species. The urgent implementation of conservation measures, guided by the age structure and reproductive characteristics of these frogs, should be consistently maintained.

## Figures and Tables

**Table 1:** Snout vent length (SVL, mm) of *Rana tavasensis* in different sex and age groups.

Age Group	Males			Age Group	Females		
	*n*	SVL			*n*	SVL	
		M ± SD	min–max			M ± SD	min–max
3	1	51.13	51.13	3	3	46.12 ± 3.43	42.33–49.02
4	1	51.97	51.97	4	1	49.87	49.87
5	2	63.24 ± 11.94	54.80–71.69	5	2	56.92± 1.29	55.48–59.51
6	2	61.50 ± 3.48	58.02–64.98	6	5	58.12 ± 0.69	56.81–59.79
7	1	66.16	66.16	7	3	61.71 ± 3.57	54.71–66.46
8	1	68.21	68.21	8	1	65.23	65.23
11	1	69.14	69.14	11	1	73.97	73.97
12	1	71.47	71.47	12	1	70.84	70.84
Total	10	62.75± 8.02	51.13–71.69	Total	17	58.04± 8.37	42.33–73.97
		**Subadult Males**				**Juveniles**	
		**SVL**				**SVL**	
		**M ± SD**	**min–max**			**M ± SD**	**min–max**
2	2	46.56 ± 5.42	42.73–50.40	1	2	23.07 ± 9.64	16.95–29.89

*n*: number of samples; M: mean; SD: standard deviation of the mean; min: minimum; max: maximum.

**Table 2:** The longevity of *Rana* populations in Türkiye assessed by skeletochronology.

Species	Location (Meter above Sea Level)	*n* (M/F)	Longevity (Years) (M/F)	References
***R. dalmatina***	Çanakkale and Kırklareli (about 200 m)	11/5	4/5	[15]
***R. dalmatina***	Devrek, Zonguldak (626 m)	17/13	6/8	[16]
***R. holtzi***	Karagöl, Niğde (2560 and 2580 m)	21/18	8/10.5	[17]
***R. holtzi***	Karagöl, Niğde (2650 m)	26/15	6/7	[11]
***R. holtzi***	Karagöl, Niğde (2558 and 2673 m)	47/42	8/9	[18]
***R. macrocnemis***	Maçka and Hıdırnebi, Trabzon Ovit, Rize Sarıkamış, Kars (350–2850 m)	100/61	8–10	[19]

*n*: number of samples; M: males; F: females.

## Data Availability

No new data were created or analyzed in this study. Data sharing is not applicable to this article.

## References

[1] Baran İ., Atatür M.K. (1986). A taxonomical survey of the mountain frogs of Anatolia. Amphib. Reptil..

[2] Veith M., Schmidtler F., Kosuch J., Baran İ., Seitz A. (2003). Paleoclimatic changes explain Anatolian mountain frog evolution: A test for alternating vicariance and dispersal event. Mol. Ecol..

[3] Franzen M., Bubmann M., Kordges T., Thiesmeier B. (2008). Die Amphibien und Reptilien der Südwest-Türkei.

[4] Ergül-Kalaycı T., Özdemir N. (2018). New locality records for Turkish endemic species *Rana tavasensis* (Baran and Atatür, 1986). J. Cryst. Growth.

[5] Çapar D., Başkale E. (2016). Population size of endemic *Rana tavasensis* in its terra typica, Turkey. Turk. J. Zool..

[6] IUCN SSC Amphibian Specialist Group *Rana tavasensis*. The IUCN Red List of Threatened Species 2023: e.T61875A100225246. https://www.iucnredlist.org/species/61875/100225246.

[7] Lande R., Engen S., Saether B.E. (2003). Stochastic Population Dynamics in Ecology and Conservation.

[8] Piferrer F., Anastasiadi D. (2023). Age estimation in fishes using epigenetic clocks: Applications to fisheries management and conservation biology. Front. Mar. Sci..

[9] Khonsue W., Matsui M., Misawa Y. (2000). Age determination by skeletochronology of *Rana nigrovittata*, a frog from tropical forest of Thailand. Zool. Sci..

[10] Smirina E.M. (1994). Age determination and longevity in amphibians. Gerontology.

[11] Guarino F.M., Erismis U.C. (2008). Age determination and growth by skeletochronology of *Rana holtzi*, an endemic frog from Turkey. Ital. J. Zool..

[12] Guarino F.M., de Pous P., Crottini A., Mezzasalma M., Andreone F. (2011). Age structure and growth in a population of *Pelobates varaldii* (Anura, Pelobatidae) from northwestern Morocco. Amphib. Reptil..

[13] Bionda C.D.L., Kost S., Salas N.E., Lajmanovich R.C., Sinsch U., Martino A.L. (2015). Age structure, growth and longevity in the common toad, *Rhinella arenarum*, from Argentina. Acta Herpetol..

[14] Başkale E., Ulubeli S.A., Kaska Y. (2018). Age structures and growth parameters of the Levantine frog, *Pelophylax bedriagae*, at different localities in Denizli, Turkey. Acta Herpetol..

[15] Genç Z., Tok C.V. (2021). A preliminary study on age determination and examination of some growth parameters in Agile Frog (*Rana dalmatina* Bonaparte, 1839) (Anura: Ranidae) specimens. Comm. J. Biol..

[16] Albayrak M., Bülbül U., Zaman E., Koç-Gür H. (2023). Life history traits in a Turkish population of the Agile frog *Rana dalmatina* Fitzinger in Bonaparte, 1839 (Anura: Ranidae). Acta Zool. Bulg..

[17] Miaud C., Üzüm N., Avcı A., Olgun K. (2007). Age, size and growth of the endemic Anatolian mountain frog *Rana holtzi* from Turkey. J. Herpetol..

[18] Yıldız M.Z., Göçmen B. (2012). Population dynamics, reproduction, and life history traits of Taurus frog, *Rana holtzi* Werner, 1898 (Anura: Ranidae) in Karagöl (Ulukışla, Niğde), Turkey. Herpetol. Rom..

[19] Kutrup B., Özdemir N., Bülbül U., Çakır E. (2011). A skeletochronological study of age, growth and longevity of *Rana macrocnemis* populations from four locations at different altitudes in Turkey. Amphibia-Reptilia.

[20] Leskovar C., Oromi N., Sanuy D., Sinsch U. (2006). Demographic life history traits of reproductive natterjack toads (*Bufo calamita*) vary between northern and southern latitudes. Amphibia-Reptilia.

[21] Castanet J., Smirina E.M. (1990). Introduction to the skeletochronological method in amphibians and reptiles. Ann. Sci. Nat. Zool..

[22] Castanet J., Francillon-Vieillot H., Meunier F.J., DeRicqlès A., Hall B.K. (1993). Bone and individual aging. Bone.

[23] Miaud C., Joly P., Castanet J. (1993). Variation in age structures in a subdivided population of *Triturus cristatus*. Can. J. Zool..

[24] Castanet J., Francillon-Vieillot H., Bruce R.C. (1996). Age estimation in desmognathine salamanders assessed by skeletochronology. Herpetologica.

[25] Odabaş Y., Bülbül U., Eroğlu A.İ., Koc H., Kurnaz M., Kutrup B. (2019). Age structure and growth in a Turkish population of the Balkan Green Lizard, *Lacerta trilineata* Bedriaga, 1886. Herpetozoa.

[26] Smirina E.M. (1974). Prospects of age determination by bone layers in Reptilia. Zool. Zhurnal.

[27] Aragón P., Fitze P.S. (2014). Geographical and temporal body size variation in a reptile: Roles of sex, ecology, phylogeny and ecology structured in phylogeny. PLoS ONE.

[28] Bülbül U., Kutrup B., Eroğlu A.İ., Koç H., Kurnaz M., Odabaş Y. (2018). Life history traits of a Turkish population of the Yellow-bellied Toad, *Bombina variegata* (Linnaeus, 1758) (Anura: Bombinatoridae). Herpetozoa.

[29] Ento K., Matsui M. (2002). Estimation of age structure by skeletochronology of a population of *Hynobius nebulosus* in a breeding season (Amphibia, Urodela). Zool. Sci..

[30] Revelle W. (2019). Psych: Procedures for Psychological, Psychometric, and Personality Research (Version 2.4.6).

[31] Pituch K.A., Stevens J.P. (2016). Applied Multivariate Statistics for the Social Sciences.

[32] Lovich J.E., Gibbons J.W. (1992). A review of techniques for quantifying sexual size dimorphism. Growth Dev. Aging.

[33] von Bertalanffy L. (1938). A quantitative theory of organic growth. Hum. Biol..

[34] Miaud C., Andreone F., Ribéron A., De Michelis S., Clima V., Castanet J., Francillon-Vieillot H., Guyétant R. (2001). Variations in age, size at maturity and gestation duration among two neighbouring popula-tions of the alpine salamander (*Salamandra lanzai*). J. Zool..

[35] Gül S., Özdemir N., Üzüm N., Olgun K., Kutrup B. (2011). Body size and age structure of *Pelophylax ridibundus* populations from two different altitudes in Turkey. Amphibia-Reptilia.

[36] Üzüm N., Avcı A., Özdemir N., Ilgaz Ç., Olgun K. (2011). Body size and age structure of a breeding population portion of the Urmia salamander, *Neurergus crocatus* Cope, 1862 (Caudata: Salamandridae). Ital. J. Zool..

[37] Arısoy A.G., Başkale E. (2019). Body size, age structure and survival rates in two populations of the Beyşehir frog *Pelophylax caralitanus*. Herpetozoa.

[38] Robson D.S., Chapman D.G. (1961). Catch curves and mortality rates. Trans. Am. Fish. Soc..

[39] Seber G.A.F. (1973). The Estimation of Animal Abundance.

[40] Patil I., Powell C. (2018). *ggstatsplot*: ‘ggplot2’ Based Plots with Statistical Details. CRAN. https://CRAN.R-project.org/package=ggstatsplot.

[41] R Core Team (2024). R: A Language and Environment for Statistical Computing, Version 4.4.1 “Race for your Life”.

[42] Guarino F.M., Angelini F., Cammarota M. (1995). A skeletochronological analysis of three syntopic amphibian species from southern Italy. Amphib. Reptil..

[43] Esteban M., Sanchiz B. (2000). Differential growth and longevity in low and high altitude *Rana iberica* (Anura, Ranidae). Herpetol. J..

[44] Miaud C., Guyétant R., Elmberg J. (1999). Variations in life-history traits in the common frog *Rana temporaria* (Amphibia: Anura): A literature review and new data from the French Alps. J. Zool. Lond..

[45] Sinsch U. (2015). Review: Skeletochronological assessment of demographic life-history traits in amphibians. Herpetol. J..

[46] Ishchenko V.G. (1996). Problems of demography and declining populations of some Euroasiatic brown frogs. Russ. J. Herpetol..

[47] Düşen O., Düşen S., Başkale E. (2019). Denizli ili Tavas Kurbağası (Rana tavasensis) tür eylem planı.

[48] Castanet J. (1994). Age estimation and longevity in reptiles. Gerontology.

[49] Yakın Y., Tok C.V. (2015). Age estimation of *Anatololacerta anatolica* (Werner, 1902) in the vicinity of Çanakkale by skeletochronology vicinity of Çanakkale by skeletochronology, *Turk*. J. Zool..

[50] Jakob C., Seitz A., Crivelli A.J., Miaud C. (2002). Growth cycle of the marbled newt (*Triturus marmoratus*) in the Mediterranean region assessed by skeletochronology. Amphib. Reptil..

[51] Smirina E.M. (1972). Annual layers in bones of *Rana temporaria*. Zool. Zh..

[52] Sinsch U., Dehling J.M. (2017). Tropical anurans mature early and die young: Evidence from eight Afromontane *Hyperolius* species and a meta-analysis. PLoS ONE.

